# Utilisation and costs of nursing agencies in the South African public health sector, 2005–2010

**DOI:** 10.3402/gha.v7.25053

**Published:** 2014-12-22

**Authors:** Laetitia C. Rispel, George Angelides

**Affiliations:** 1 Centre for Health Policy & Medical Research Council Health Policy Research Group, School of Public Health, Faculty of Health Sciences, University of the Witwatersrand, Johannesburg, South Africa; 2 Gauteng Department of Health, Johannesburg, South Africa

**Keywords:** nursing agency, costs, utilisation, health workforce, South Africa

## Abstract

**Background:**

Globally, insufficient information exists on the costs of nursing agencies, which are temporary employment service providers that supply nurses to health establishments and/or private individuals.

**Objective:**

The aim of the study was to determine the utilisation and direct costs of nursing agencies in the South African public health sector.

**Design:**

A survey of all nine provincial health departments was conducted to determine utilisation and management of nursing agencies. The costs of nursing agencies were assumed to be equivalent to expenditure. Provincial health expenditure was obtained for five financial years (2005/6–2009/10) from the national Basic Accounting System database, and analysed using Microsoft Excel. Each of the 166,466 expenditure line items was coded. The total personnel and nursing agency expenditure was calculated for each financial year and for each province. Nursing agency expenditure as a percentage of the total personnel expenditure was then calculated. The nursing agency expenditure for South Africa is the total of all provincial expenditure. The 2009/10 annual government salary scales for different categories of nurses were used to calculate the number of permanent nurses who could have been employed in lieu of agency expenditure. All expenditure is expressed in South African rands (R; US$1 ∼ R7, 2010 prices).

**Results:**

Only five provinces reported utilisation of nursing agencies, but all provinces showed agency expenditure. In the 2009/10 financial year, R1.49 billion (US$212.64 million) was spent on nursing agencies in the public health sector. In the same year, agency expenditure ranged from a low of R36.45 million (US$5.20 million) in Mpumalanga Province (mixed urban-rural) to a high of R356.43 million (US$50.92 million) in the Eastern Cape Province (mixed urban-rural). Agency expenditure as a percentage of personnel expenditure ranged from 0.96% in KwaZulu-Natal Province (mixed urban-rural) to 11.96% in the Northern Cape Province (rural). In that financial year, a total of 5369 registered nurses could have been employed in lieu of nursing agency expenditure.

**Conclusions:**

The study findings should inform workforce planning in South Africa. There is a need for uniform policies and improved management of commercial nursing agencies in the public health sector.

The global quest for universal health coverage has once again put the spotlight on the centrality of the health workforce to achieving this goal (1). New modelling estimates suggest a global deficit of around 12.9 million nurses, midwives, and physicians by 2035 (1). In 2010, it was estimated that there were more than 100,000 vacancies – a proxy for staff shortages – for all categories of health care professionals in the South African public health sector (2). Nurses constituted the vast majority of these vacancies (2).

Worldwide, the management of nursing shortages in health facilities is a major problem (3). In Australia, Canada, the United Kingdom, and the United States of America, temporary nursing staff (e.g. agency or casual nurses) have been used to fill the gap created by a lack of full-time, permanent nurses in the health care system (3–8). In South Africa, anecdotal evidence suggests a growth in commercial nursing agencies, which are temporary employment service providers that supply different categories of nurses to health establishments and/or private individuals (9). A large, representative cross-sectional study conducted in 2010 found that one in every 11 nurses worked for a commercial nursing agency and that 37.8% of study participants engaged in agency nursing in the year preceding the survey (10). These agency nurses are employed through nursing agencies.

Much of the literature on nursing agencies is concerned with high-income countries (5, 6, 8, 11–16). A key focus and concern of the literature have been on the management of temporary or agency staff (11, 12, 14, 15, 17, 18), or the negative implications of temporary staff for nursing managers, communication, patient care, and/or health service delivery (19–21). In recent years, Canada, the United Kingdom, and the United States have studied their nursing workforce profiles, including the number of casual and/or agency nurses, in an attempt to assist with improved health workforce planning and forecasting (22–25). The UK National Audit Office has a monitoring system on the use and management of all temporary nursing staff in acute hospital and foundation trusts, and trend information is available over a period of time (18, 26, 27).

There are few empirical studies on nursing agencies (3, 6, 8), and these tend to be descriptive, sometimes lacking the methodological rigour needed to make generalisable conclusions. Globally, insufficient information exists on the costs of agency nursing. The UK National Audit Office has commissioned several studies, including a census of National Health Service (NHS) Trusts to examine the demand for temporary staff (including agency staff), their utilisation, the costs of procurement, and the impact of initiatives to improve quality and expenditure information (18, 27). A Melbourne study on agency nursing found high utilisation of agency nurses in response to problems of recruitment and retention of nurses in specialty areas, busy and fluctuating caseload units, and lack of permanent night duty staff (8). However, there are methodological limitations as it was a telephone survey, financial data were posted, and the agency response rate was poor (8). In South Africa, expenditure information is more readily available, particularly in the private health sector (28), but the costs of nursing agencies in the public sector have not been well researched.

Cost information is important for the management of temporary staff in a health facility, for shaping policy responses, monitoring trends, and/or benchmarking the performance of nursing agencies in the health system (29, 30). Costing data also allow for improved workforce planning, including comparisons of quality and performance between agency staff and permanent staff (26). In light of this and the dearth of empirical information, the aim of this study was to determine utilisation and costs of nursing agencies in the South African public health sector.

## Methodology

The study setting was the nine provincial health departments in South Africa, which were classified as follows: urban (Gauteng and Western Cape), rural (Limpopo, Northern Cape, and North West), and mixed urban-rural (Eastern Cape, Free State, KwaZulu-Natal, and Mpumalanga). The study was approved by the Human Research Ethics Committee (Medical) of the University of the Witwatersrand in Johannesburg. The provincial health authorities also provided approval for the study.

A *survey* was conducted among all nine provincial health departments in South Africa to determine the utilisation and management of nursing agencies. The survey aimed to develop an understanding of the drivers of agency nurse utilisation and to explore the policies on, and management of, nursing agencies. Four face-to-face interviews and three telephonic interviews were conducted, using a semi-structured interview schedule. In the case of one province, the human resource manager in the province completed the interview schedule and faxed it back to the researchers. The interview schedule focussed on utilisation of nursing agencies in the province; the type of health facilities or services that utilise agency nurses; policy frameworks that guide the use of nursing agencies, including the existence of a specific provincial policy; and the perceived advantages and disadvantages of nursing agencies. The information from the interviews was coded and analysed using thematic content analysis.

A cost analysis of nursing agencies was done for the 5-year period from 2005 until 2010. We assumed that the direct costs of nursing agencies were equivalent to *expenditure* on nursing agencies. Data on provincial health expenditure were obtained for five financial years (2005/6–2009/10) from the national transversal Basic Accounting System (BAS) database (i.e. official government statistics). The financial year in government commences on 1 April each year and ends on 31 March of the following year. The data were exported into Microsoft Excel in order to facilitate uniform coding of the expenditure information and to do trend analysis across the entire period (2005/06 to 2009/10). Each financial year was put into a separate worksheet. The number of expenditure line items ranged from 30,595 in the 2005/6 financial year to 34,978 in the 2009/10 financial year, totalling 166,466 line items for coding over the 5-year period. Each line item was coded meticulously, depending on the type of expenditure, and similar items were grouped and aggregated. The coding also took into account the province or health facility where the expenditure occurred. Once each item was coded, cross-tabulations and calculations were done to ensure that the same total expenditure was obtained for each provincial health department, thus ensuring both validity and reliability of the study results.

Overall provincial health expenditure consists of all expenditure items, including compensation of employees, goods and services (operational expenditure), transfer payments (to municipalities or non-governmental organisations), and capital expenditure. Personnel expenditure, also called compensation of employees, consists of salaries, staff benefits, and overtime payments. Nursing agency expenditure consists of all expenses paid to commercial nursing agencies (including administration fees) and is paid from the goods and services budget of each provincial health department.

For each of the nine provinces, the total personnel and nursing agency expenditure was calculated for each financial year. Agency nursing expenditure as a percentage of the total personnel expenditure was then calculated, for each province and for each financial year. The nursing agency expenditure for all provinces was totalled to arrive at agency expenditure for South Africa for each financial year, and over the 5-year period.

In order to calculate the number of full-time-equivalent (FTE) nurses who could have been employed for the given nursing agency expenditure for that province, the 2009/10 government salary scales for different categories of nurses (professional, enrolled, and auxiliary nurses) were obtained. The median salary for each nursing category was calculated. Assuming standard benefits such as health insurance and housing subsidy, 30% was added to the amount. This amount for each nursing category was then used to calculate the number of FTE nurses. All expenditure is expressed in South African rands (R; US$1 ∼ R7, 2010 prices).

## Results

### Utilisation of nursing agencies, policies and management

Eight out of nine provincial health departments responded to the provincial survey on nursing agencies (89% response rate). The team tried unsuccessfully for 18 months to get a response from the Northern Cape Provincial Health Department.

Table 1 shows the reported utilisation of nursing agencies in the South African public health sector.

**Table 1:** Utilisation and management of nursing agencies in the South African public health sector

Province	Nursing agency utilisation	Existence of specific provincial health policy framework	Advantages	Disadvantages
Eastern Cape	Yes, in tuberculosis and public-private partnership hospitals	Unsure	Agencies deploy staff to geographical areas or health facilities where it is difficult to find staff.	Agencies not well regulated Many agency nurses not registered with the South African Nursing Council
Free State	Yes	Unsure	Staff supplementation Savings on personnel budget as they are paid from the goods and services budget	Agencies do not send nurses with required skills. Some agency nurses not familiar with the public health sector. Nurses abuse the system by working through agencies while on leave at the primary employer.
Gauteng	Yes, contract with 10 agencies Mostly used in large hospitals and specialised areas (e.g. critical care)	Yes – provincial nursing agency policy	Assist with staff shortages in specialty areas, such as critical care units	Managers use nursing agencies to manage absenteeism. Agencies send inexperienced nurses or nurses without specialised skills. Contractual obligations not enforced by hospital management. Possible corruption and collusion between agencies, managers, and individual nurses
Kwazulu-Natal	Stopped utilisation of nursing agencies due to budgetary constraints	No Used government supply chain management policy	–	Nurses employed by provincial Department of Health moonlighted via agencies in their hospitals of employment.
Limpopo	No	Not applicable	Not applicable	Not applicable
Mpumalanga	Yes, contract with 2 agencies	No	Able to provide efficient services	Employees compare the wages received from agencies with departmental salaries, and that creates conflict.
North West	No	Not applicable	Not applicable	Not applicable
Western Cape	Yes, contract with 9 agencies Mostly used in large hospitals and specialised areas	Yes, public sector policies on remunerative work outside the public service and provincial human resource management	Able to provide staff at short notice	High costs Inexperienced nurses Suboptimal quality of care

The provincial survey (Table 1) found that budgetary constraints have either prevented the use of nursing agencies (North West, Eastern Cape, and Limpopo) or led to the termination (KwaZulu-Natal) or the restricted and/or controlled use of nursing agencies (Free State, Gauteng, Mpumalanga, and Western Cape). Gauteng and Western Cape Provinces reported specific policy frameworks that guide the utilisation of nursing agencies. In Free State and Mpumalanga, the generic government procurement policy was used. The perceived advantages of utilising nursing agencies were that they complemented the existing number of staff, especially in specialised areas, and cost savings on the personnel budget, particularly after the implementation of the occupation specific dispensation (OSD), a financial incentive for health professionals in the public service (31). Reported disadvantages of nursing agencies were: the failure of agencies to supply the required nursing skills; inappropriate use of agencies, often to manage permanent staff absenteeism; lack of enforcement of contractual obligations; possible corruption and collusion between agencies, managers, and nurses; and potential abuse of the system when provincial government nurses do moonlighting through an agency.

### Total nursing agency expenditure in the South African public health sector, 2005–2010

**Fig. 1:**
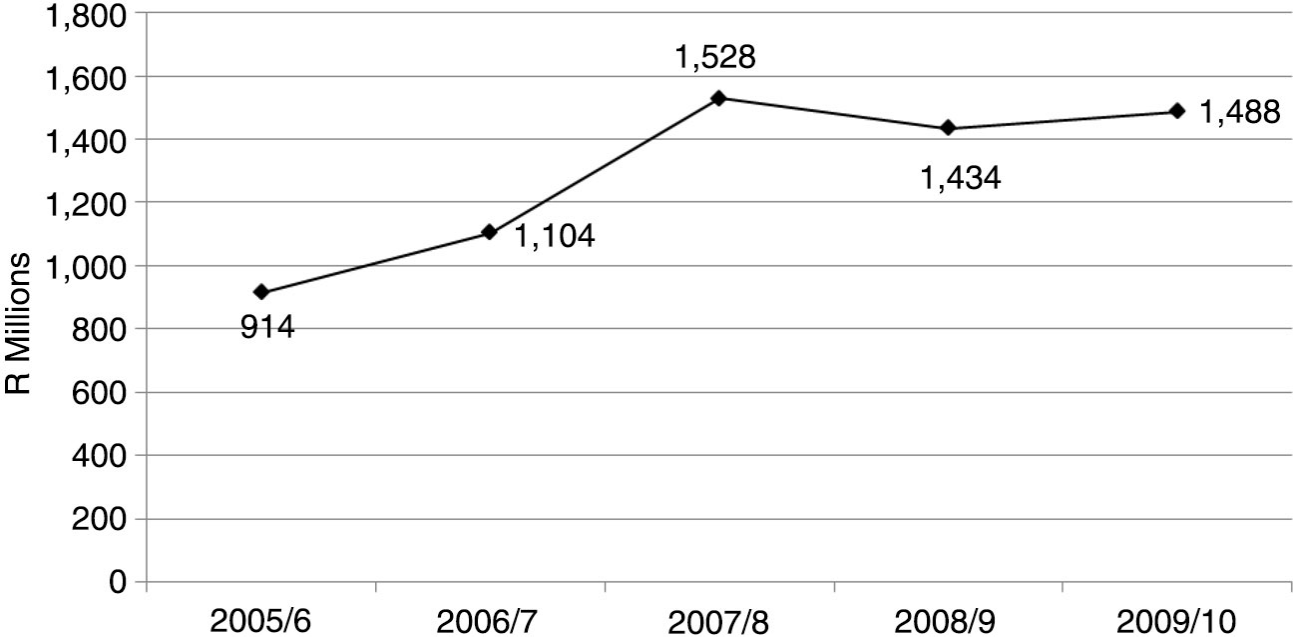
Trends in total nursing agency expenditure in the South African public health sector, 2005–2010. Source: South Africa National Treasury, national transversal Basic Accounting System (BAS). R = South African rands.

Figure 1 shows the overall nursing agency expenditure in the South African public health sector. Expenditure ranged from R914.29 million (US$130.61 million) in the 2005/6 financial year to a peak of R1.53 billion (US$218.22 million) in 2007/8. In the 2009/10 financial year, R1.49 billion (US$212.64 million) was spent on nursing agencies. The total amount spent on nursing agencies in the South African public health sector for the 5-year study period was R6.47 billion (US$924.01 million).

### Trends in nursing agency expenditure

**Fig. 2:**
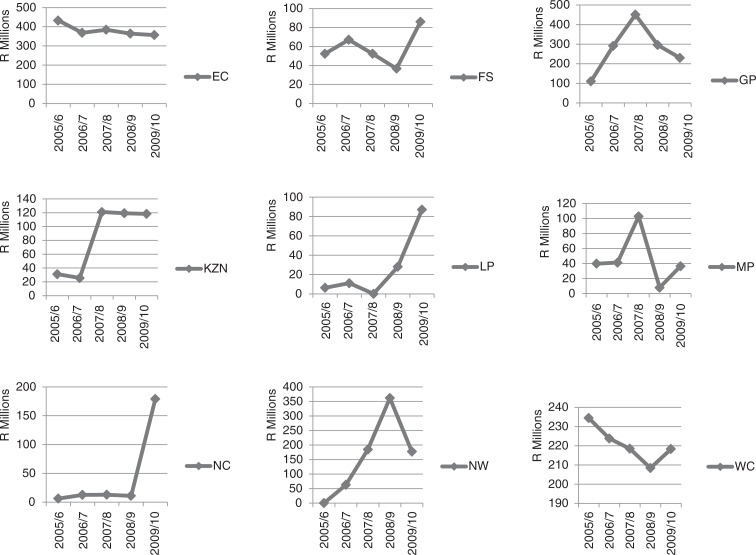
Trends in agency nursing expenditure by province, 2005–2010. Source: South Africa National Treasury, national transversal Basic Accounting System (BAS). EC = Eastern Cape; FS = Free State; GP = Gauteng; KZN = KwaZulu Natal; LP = Limpopo; MP = Mpumalanga; NC = Northern Cape; NW = North West; WC = Western Cape; R = South African rands.

Table 2 and Fig. 2 show the trends in nursing agency expenditure for each of the nine provinces over the 5-year period. As can be seen from Table 2, in 2009/10, agency expenditure ranged from a low of R36.45 million (US$5.21 million) in Mpumalanga Province to a high of R356.43 million (US$50.92 million) in the Eastern Cape Province. Fig. 2 shows that there were erratic expenditure patterns in the majority of provinces, and unexplained peaks in Gauteng in 2007/8, in North West in 2008/9, and in Northern Cape in 2009/10.

**Table 2:** Trends in nursing agency expenditure in the South African public health sector, 2005–2010.

Province	2005/6	2006/7	2007/8	2008/9	2009/10
Eastern Cape	432,799,564	368,740,527	384,777,196	364,378,884	356,429,449
Free State	52,395,839	67,101,223	52,417,250	36,769,318	85,968,276
Gauteng	110,972,166	291,240,036	450,566,358	296,302,273	229,782,134
KwaZulu-Natal	31,192,138	25,542,244	121,060,152	119,355,192	118,437,227
Limpopo	6,354,945	11,098,524	78,030	28,030,416	87,177,905
Mpumalanga	39,818,398	41,153,298	102,911,333	7,859,761	36,445,893
Northern Cape	6,318,734	12,557,108	12,961,058	11,042,380	178,919,686
North West	0	62,684,538	184,306,034	361,680,916	177,014,047
Western Cape	234,437,117	223,761,383	218,497,181	208,431,408	218,279,792
TOTAL	914,288,902	1,103,878,880	1,527,574,592	1,433,850,547	1,488,454,407

Source: South Africa National Treasury, national transversal Basic Accounting System (BAS).

### Nursing agency expenditure as a percentage of total provincial health expenditure

Table 3 shows trends in nursing agency expenditure as a percentage of total provincial health expenditure.

**Table 3:** Trends in nursing agency expenditure as a percentage of overall provincial health expenditure

Province	2005/6 (%)	2006/7 (%)	2007/8 (%)	2008/9 (%)	2009/10 (%)
Eastern Cape	6.62	4.73	4.77	3.45	2.93
Free State	1.69	1.99	1.36	0.83	1.63
Gauteng	1.04	2.43	3.29	1.80	1.19
KwaZulu-Natal	0.29	0.21	0.79	0.68	0.53
Limpopo	0.14	0.19	0.00	0.35	0.96
Mpumalanga	1.44	1.30	2.84	0.18	0.63
Northern Cape	0.57	0.95	0.64	0.63	8.06
North West	0.00	4.64	4.79	8.14	3.44
Western Cape	3.97	3.38	2.82	2.33	2.03

Source: South Africa National Treasury, national transversal Basic Accounting System (BAS).

As can be seen from Table 3, in Limpopo (rural) and KwaZulu-Natal (mixed urban-rural) provinces, nursing agency expenditure was less than 1% of overall health spending for the entire study period. Both Eastern Cape (mixed) and Western Cape (urban) showed a 50% decline in nursing agency expenditure as a percentage of overall health spending between the beginning and end of the study period. In Gauteng (urban), there were increases between 2005/6 and 2007/8, with declines in the 2008/9 and 2009/10 financial years. Free State (mixed) appears to have managed to keep nursing agency expenditure to less than 2% of overall health spending. There were large fluctuations in the rural provinces of Northern Cape and North West, where nursing agency expenditure as a percentage of overall provincial spending was 8.06% in the Northern Cape in 2009/10 and 8.14% in North West in 2008/9.

### Nursing agency expenditure as a percentage of personnel expenditure

Table 4 illustrates trends in nursing agency expenditure as a percentage of personnel expenditure in each province for the study period.

**Table 4:** Nursing agency expenditure as a percentage of personnel expenditure

Province	2005/6 (%)	2006/7 (%)	2007/8 (%)	2008/9 (%)	2009/10 (%)
Eastern Cape	12.56	9.56	8.40	6.02	4.89
Free State	2.82	3.33	2.23	1.29	2.68
Gauteng	2.38	5.36	6.90	3.61	2.28
KwaZulu-Natal	0.52	0.38	1.39	1.19	0.96
Limpopo	0.23	0.34	0.00	0.60	1.63
Mpumalanga	2.67	2.40	5.15	0.31	1.23
Northern Cape	1.21	1.84	1.63	1.25	11.96
North West	0.00	6.75	–a	15.13	6.44
Western Cape	7.86	6.53	5.27	4.28	3.89

a Unable to calculate because of missing data.

Source: South Africa National Treasury, national transversal Basic Accounting System (BAS).

Eastern Cape and Western Cape Provinces show more than 50% declines in nursing agency expenditure as a percentage of personnel expenditure over the study period. In Northern Cape, nursing agency expenditure constituted 11.96% of personnel spending in the 2009/10 financial year, whereas in North West, agency expenditure was 15.13% of the personnel expenditure in the 2008/9 financial year. In Gauteng, nursing agency expenditure as a percentage of personnel spending showed a similar pattern to overall spending, with increases between 2005/6 and 2007/8, and declines in the 2008/9 and 2009/10 financial years. There was no consistent spending pattern in the other provinces.

### Opportunity costs of nursing agency expenditure

In 2009/10, the annual salary package of a professional nurse (4 years of training) was R277,226 (US$40,000), an enrolled nurse (2 years of training) earned an annual salary of R154,471 (US$22,000), and an auxiliary nurse (1 year of training) earned an annual salary of R119,646 (US$17,000). Using these salary packages, Table 5 shows the number of nurses of each category who could have been employed in lieu of nursing agency expenditure for the 2009/10 financial year.

**Table 5:** Opportunity costs of nursing agency expenditure, 2009/10

Province	Professional nurses	Enrolled nurses	Auxiliary nurses
Eastern Cape	1286	2307	2979
Free State	310	557	719
Gauteng	829	1488	1921
Kwazulu-Natal	427	767	990
Limpopo	314	564	729
Mpumalanga	131	236	305
Northern Cape	645	1158	1495
North West	639	1146	1479
Western Cape	787	1413	1824
South Africa	5369	9636	12,441

Data sources: South Africa National Treasury, national transversal Basic Accounting System (BAS). Annual salary packages for different categories of nurses obtained from the Department of Public Service and Administration.

As can be seen from Table 5, in 2009/10, a total of 5369 professional (registered) nurses could have been employed in the South African public health sector. Alternatively, nursing agency expenditure could have funded 9636 enrolled nurses or 12,441 auxiliary nurses.

## Discussion

The provincial survey revealed wide variations in the utilisation and management of nursing agencies across the nine provinces (Table 1), despite the growth in public sector staff numbers in the last decade (32). In the survey, North West and Limpopo Provinces reported that there was no utilisation of nursing agencies, and therefore no need for policies and/or the management of these agencies. However, this was contradicted by the costing study as expenditure was recorded in all provincial health departments. These findings on utilisation could reflect a lack of knowledge on the part of the respondents who were interviewed. On the other hand, the findings point to the disjuncture and lack of communication between human resource and finance departments at the head offices of provincial health departments, and between the head offices (centre) and lower level health facilities. Other studies have also pointed to the problems of suboptimal communication and ‘silo’ functioning both among and within provincial health departments in South Africa (32, 33).

The costing study found that nursing agency expenditure in the South African public sector ranged from R914.29 million (US$130.61 million) in the 2005/6 financial year to R1.49 billion (US$212.64 million) in the 2009/10 financial year. This total amount of R6.47 billion (US$924.01 million) that was spent on nursing agencies for the 5-year study period was more than the 2009/10 provincial health budget of Free State, Mpumalanga, Northern Cape, or North West Province (34). We could not find comparable studies of nursing agency expenditure in other low- and middle-income settings. In England, a 2004/5 census of temporary nursing staff in acute NHS hospital and foundation Trusts found that the Trusts spent a total amount of £238 million (pounds Sterling) on *agency* nursing staff during 2004/5, and £773 million on all temporary nursing staff (35). However, there are vast differences in the organisation of the health care system, staffing needs, the quantum of health budgets, and/or health expenditure between England and South Africa; hence, the findings are not comparable.

The costing study found that each of the nine provincial health departments spent considerable sums of money on nursing agencies that supply temporary staff (Table 2 and Fig. 2). There were large variations in nursing agency expenditure across the provincial health departments as well as in nursing agency expenditure as a percentage of total provincial health expenditure (Table 3). The latter ranged from 0.53% in KwaZulu-Natal to 8.06% in the Northern Cape Province. These geographical variations in expenditure on temporary nursing staff were also found in the 2006 report of the National Audit Office in England (18) and a more recent newspaper investigation on NHS expenditure on temporary nursing staff (36).

In the 2009/10 financial year, nursing agency expenditure as a percentage of personnel expenditure ranged from 0.96% in KwaZulu-Natal to 11.96% in Northern Cape. The reasons for the variations in expenditure are not clear. In the provincial survey, KwaZulu-Natal indicated that budgetary constraints prevented them from utilising nursing agencies. This could explain the relatively low percentage of nursing agency expenditure, relative to personnel spending. Northern Cape did not respond to the survey, thus its reasons for the utilisation of nursing agencies could not be elicited. A study in Melbourne, Australia, found that expenditure on agency staff ranged from 5 to 10% of the monthly staffing budget, but this figure was as high as 35% in some institutions (37). In England, nursing expenditure as a percentage of the nursing workforce expenditure was 3% in 2004/5 (18). Notwithstanding the report from North West Province that they did not utilise nursing agencies, in the 2008/9 financial year, nursing agency expenditure constituted 15.13% of the personnel expenditure. This was the highest proportion of provincial personnel spending on temporary or casual staff. There are several possible reasons that could explain this figure. Firstly, it could be a financial transaction error on the part of the North West Province, despite the stringent requirements of the Public Finance Management Act (38). Secondly, the utilisation of nursing agencies could be an indication of staff shortages in the many rural health facilities in the province. This reason is corroborated by an estimated 743 vacancies for all categories of nurses in 2010 (2). Thirdly, nursing agencies are paid from the goods and services budget, and not from the personnel budget. Consequently, the spending on nursing agencies could have been a mechanism to prevent or hide overspending on the personnel budget.

There are several limitations of the study. In the costing study, we assumed that direct costs were equal to expenditure. We also used public sector vacancies as a proxy for staff shortages, which is reasonable in the absence of national staffing norms. However, there is no standard definition of a ‘vacancy’, and in some instances a vacancy could be measured against an old, outdated organisational structure or against the available budget (32). Furthermore, the national human resource plan was based on modelling estimates, and there was no empirical assessment of health workforce requirements against health need, itself a contested concept (33). Thirdly, we assumed a zero-sum trade-off between nurse agency costs and permanent staff employment, which may not hold in all instances. For example, agency nurses could be used to manage the cost of variations in patient demand, thus resulting in efficiency gains because of the reduction in fixed salary costs. We also do not have comparative data on the expenditure on a full-time vacancy filled by the public sector and the expenditure on a part-time equivalent filled from the agency.

The study also relied on self-reported information from senior government officials, which was shown to be inaccurate in at least two of the provinces. Although financial statistics tend to be more accurate than other information, the study used official government statistics that the provincial health departments submitted to the National Treasury, and data quality remains a challenge.

Notwithstanding these limitations, the study has numerous strengths. The study is novel in combining and contrasting information obtained through a provincial survey, with a detailed analysis and interpretation of expenditure on nursing agencies, and the trends in such expenditure over a 5-year period. A major strength of the study is its national focus, thus making an important contribution to the knowledge and understanding of the expenditure, management, and utilisation of nursing agencies in the South African public health sector. The study provides empirical evidence of the extensive utilisation of temporary agency staff in the South African public health sector, which is illustrated by the high expenditure on nursing agencies in all provinces. An important finding of the study is that 5369 professional or registered nurses with 4 years of training could have been employed in the South African public health sector in 2009/10 in lieu of nursing agency expenditure. The study also illustrates the usefulness of a trend analysis of routine financial data, and the potential of such analysis to inform policy or decision making.

Notwithstanding these limitations, the study has numerous strengths. The study is novel in combining and contrasting information obtained through a provincial survey, with a detailed analysis and interpretation of expenditure on nursing agencies, and the trends in such expenditure over a 5-year period. A major strength of the study is its national focus, thus making an important contribution to the knowledge and understanding of the expenditure, management, and utilisation of nursing agencies in the South African public health sector. The study provides empirical evidence of the extensive utilisation of temporary agency staff in the South African public health sector, which is illustrated by the high expenditure on nursing agencies in all provinces. An important finding of the study is that 5369 professional or registered nurses with 4 years of training could have been employed in the South African public health sector in 2009/10 in lieu of nursing agency expenditure. The study also illustrates the usefulness of a trend analysis of routine financial data, and the potential of such analysis to inform policy or decision making.

The study findings have implications for quality of care, human resource planning and management, and health policy development in South Africa. The 2010 national core standards (39) and the 2013 promulgation of the National Health Amendment Act underscore the importance of quality of care in the country (40). The domain of *patient safety, clinical governance, and clinical care* in the national core standards focusses on ‘how to ensure quality nursing and clinical care and ethical practice, and reduce unintended harm to health care users or patients in identified cases of greater clinical risk’ (39, p. 11). Although the utilisation of temporary agency nurses is a short-term solution to staffing shortages, there is a growing body of evidence that casual or temporary staffing contributes to poor quality of patient care (41–44). Several US studies have found statistically significant associations between the employment of agency nurses and health care deficiencies in nursing homes (41), hospital medication errors (43), and the risk of bloodstream infections among patients with central venous catheters in intensive care units (44). In the United Kingdom, it was also found that temporary staffing could undermine the quality of patient care (18). However, a study of 605 UK general and specialist wards between 2004 and 2009 found no differences in quality scores between temporary and permanent nursing staff (45). Nonetheless, the UK Department of Health has stated that replacing temporary staff with experienced permanent staff leads to increased productivity and better patient care (18). In South Africa, we could not find any studies on the association between the use of agency nurses and quality of care. However, a 2010 survey found high moonlighting rates in critical care units in both the public and private health sectors (10). This implies that temporary nurses are utilised extensively in hospital critical care units in South Africa. Hence, similar issues of quality of care could be found in South Africa.

The management of human resources is a key aspect of quality of care as it enables the delivery of safe and effective patient care (1, 39). Hence, the study findings also have implications for human resource planning and management. The provincial survey found that there is no national policy on the management and utilisation of nursing agencies. There is global evidence that hiring agency nurses costs up to three times more than permanent staff (18, 23, 24, 27, 45). Although the utilisation of temporary staff is influenced by efficiency measures, staff shortages, and changing patient numbers and acuity, the demand for temporary nursing staff is also shaped by poor staff planning, staff absenteeism, and lack of involvement of nurses in decision making (3, 27, 45, 46).

In South Africa, there is a policy vacuum on commercial nursing agencies. Until 2005, these agencies were required to register with the South African Nursing Council (9), but the National Health Act classified nursing agencies as health establishments under the jurisdiction of the Department of Health (47). Although the 5-year strategy on nursing education, training, and practice, launched in 2013, recommended the urgent development and implementation of regulations for nursing agencies (48), the draft regulations were vague and unlikely to lead to better control of nursing agencies. The progress has stalled. The experience of regulating nursing agencies in other countries such as the United Kingdom is instructive. In that country, minimum standards and regulations on nursing agencies aim to ensure ethically and legally compliant service delivery (26). The UK national nursing association has developed a complementary set of guidelines for employers, nursing agencies, and agency nurses that summarise regulatory requirements and induction processes to meet minimum standards (49, 50).

Both the South African 5-year strategic plans on human resources for health (2) and on nursing education, training, and practice (36) recommend policy development or improved management of nursing agencies, albeit in a cursory manner. The finalisation of regulations on commercial agencies and the development of good practice guides are critical. Such regulations and practice guides could draw from the UK experience (18, 26) and must address issues of planning for the use of agency nurses, controlling demand, appointing nursing agencies, using temporary nursing staff in an efficient and effective manner, and monitoring expenditure and the performance of all nurses.

## Conclusions

This study has highlighted the need to plan strategically for the use of temporary agency nurses, and to monitor nursing agency expenditure, as part of a comprehensive workforce plan. The planned health reforms towards universal health coverage in South Africa necessitate proactive health workforce planning to improve population health, ensure the delivery of quality care, and align expenditure to budgets.

Notwithstanding the size and complexity of the health sector, implementation of nursing agency regulations and good practice guides must be a priority for the chief nursing officer. This is in light of the well-described gaps between policy and legislation on the one hand, and implementation and practice translation on the other hand (33, 51). Although the development of legislation, policies, and guidelines is important, the Health Ministry should also pay sufficient attention to the management of change, and ensure consultation with the provincial health departments (as implementing agencies) and front-line nurses. Finally, on-going monitoring of nursing agencies is important, thereby enhancing public accountability.
